# Obesity and hypoxia have differential effects on myocardial innervation in the right ventricle of the male mouse heart

**DOI:** 10.1111/joa.14221

**Published:** 2025-01-18

**Authors:** Louisa‐Chiara Mierswa, Julia Schipke, Christian Mühlfeld

**Affiliations:** ^1^ Hannover Medical School Institute of Functional and Applied Anatomy Hannover Germany; ^2^ Biomedical Research in Endstage and Obstructive Lung Disease Hannover (BREATH) Member of the German Center for Lung Research (DZL) Hannover Germany

**Keywords:** autonomic nervous system, chronic hypoxia, obesity, right ventricle

## Abstract

Obesity, along with hypoxia, is known to be a risk factor for pulmonary hypertension (PH), which can lead to right ventricular hypertrophy and eventually heart failure. Both obesity and PH influence the autonomic nervous system (ANS), potentially aggravating changes in the right ventricle (RV). This study investigates the combined effects of obesity and hypoxia on the autonomic innervation of the RV in a mouse model. Male C57BL/6N mice were subjected to a control diet (CD) or a high‐fat diet (HFD) for 30 weeks, with subsets of the mice exposed to chronic normobaric hypoxia (13% O_2_) during the final 3 weeks. Light and electron microscopic stereology was used to quantify various parameters of nerve fibres innervating the RV myocardium. HFD‐induced obesity significantly increased the total length of nerve fibres and axons in the RV under normoxic conditions, indicating hyperinnervation. Quantitatively, the length density of nerve fibres per unit volume of RV (unit: x10^‐3^ µm^‐2^) was similar in CD (0.158 ± 0.04), CD‐Hyp (0.176 ± 0.06) and HFD‐Hyp (0.147 ± 0.05). In contrast, in HFD the length density of nerve fibres showed higher values 0.206 ± 0.054. The total length of nerve fibres increased by 67% from 2.61 m ± 0.77 m in CD to 4.37 m ± 1.51 m in HFD. The total length of axons increased by 80% from 8.87 m ± 2.75 m to 15.95 m ± 4.62 m. However, when obesity was combined with hypoxia, the total axon length was significantly reduced by 27% in HFD‐Hyp compared with HFD. In addition, the mean number of axon profiles per nerve fibre profile decreased from 3.44 ± 0.68 in HFD to 2.95 ± 0.43 in HFD‐Hyp. Interestingly, chronic hypoxia alone did not significantly alter RV innervation but led to RV hypertrophy, independent of the diet. The attenuation of obesity‐induced hyperinnervation by hypoxia suggests a complex and potentially antagonistic interaction between these conditions. In conclusion, obesity induced by a HFD caused hyperinnervation of the RV, whereas chronic hypoxia alone did not significantly alter RV innervation. Surprisingly, chronic hypoxia attenuated the obesity‐induced changes in RV innervation. These findings indicate that the effects of obesity and hypoxia‐induced PH on RV innervation are distinct and potentially antagonistic.

## Graphical abstract

Obesity induced by a high fat diet caused hyperinnervation of the right ventricle, whereas chronic hypoxia alone did not significantly alter right ventricular innervation. Surprisingly, chronic hypoxia attenuated the obesity‐induced changes in right ventricular innervation. These findings indicate that the effects of obesity and hypoxia‐induced pulmonary hypertension on right ventricular innervation are distinct and potentially antagonistic.

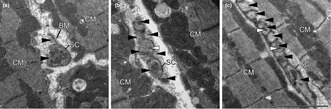

## 1 INTRODUCTION

The global prevalence of obesity has significantly increased over recent decades, presenting a substantial public health concern. In 2022, 2.5 billion adults were classified as overweight, with 890 million identified as obese (World Health Organization, 2024). Obesity is associated with cardiovascular diseases such as atherosclerosis, heart failure and pulmonary hypertension (PH), with the latter posing a particular risk to the right ventricle (RV) (Vonk Noordegraaf et al., 2019). Pathologically, both interstitial and cardiomyocyte fat accumulation occur and result in cardiomyocyte degeneration and apoptosis (Poirier et al., 2006). Hemodynamically, excess adipose tissue increases total blood volume, stroke volume and hence cardiac output, thus leading to ventricular dilatation, hypertrophy and dysfunction (Alpert, 2001; Alpert & Hashimi, 1993; Lavie et al., 2009). Moreover, obesity can also lead to obstructive sleep apnea/hypoventilation syndrome, resulting in severe episodes of hypoxia and pulmonary arteriolar vasoconstriction, which in turn causes PH (Alpert & Hashimi, 1993). This condition increases the workload on the RV, leading to hypertrophy and dysfunction (Bagheri et al., 2023). The autonomic nervous system (ANS) of the RV plays a decisive role in the regulation of cardiac function. The sympathetic innervation of the RV is mainly mediated via the ganglia stellata, with nerve fibres entering the myocardium via the epicardium (Govoni et al., 2011; Vaseghi & Shivkumar, 2008). Sympathetic activity leads to positive inotropy, chronotropy and dromotropy (Florea & Cohn, 2014) via stimulation of ß1 and ß2 adrenoreceptors by noradrenaline. Increased sympathetic activity leads to higher cardiac output and increased lipolysis and gluconeogenesis as well as inhibited insulin secretion (Lambert et al., 2010). In contrast to the sympathetic nervous system, the parasympathetic innervation of the ventricles is sparse (Vaseghi & Shivkumar, 2008).

Various obesity‐related cardiovascular diseases, such as hypertension or heart failure, can lead to changes in the function of the ANS (Florea & Cohn, 2014), with chronic sympathetic overactivity leading to overstimulation of β‐adrenergic receptors. This may cause structural and functional changes in the RV. In addition, excessive fat accumulation leads to increased production of proinflammatory cytokines, which cause a chronic pro‐inflammatory milieu. Previous studies have shown that this in turn can lead to a dysregulation of the sympathetic innervation of the heart and thus impair the adaptability of the heart muscle (Lambert et al., 2015).

Kondo et al. showed in a rat model that stroke‐prone, spontaneously hypertensive rats (SHRSP) had a higher density of noradrenergic nerve fibres in the myocardium of the RV than normotensive Wistar‐Kyoto (WKY) rats. This hyperinnervation indicates primary changes that precede the development of hypertension (Kondo et al., 1996). In another study on rats (Aubin et al., 2010), eight‐week high‐fat diet led to increased expression of tyrosine hydroxylase in the left ventricle (LV), the key enzyme of catecholamine synthesis. In addition, chronic hypoxia is associated with increased sympathetic activity (Calbet, 2003; Swenson, 2020) as indicated by studies that have measured the concentration of catecholamines in the blood and urine of human individuals (Richalet et al., 2024).

The present study therefore tested the hypothesis that obesity and hypoxia cause changes of the RV innervation. In particular, it was hypothesised that obesity causes RV hyperinnervation which is aggravated by chronic hypoxia as a second hit.

## 2 MATERIALS AND METHODS

### 2.1 Animals and tissue preparation

All animal experiments were carried out in accordance with the European Directive 2010/63/EU and authorized by the Lower Saxony State Office for Consumer Protection (LAVES, file no. 18/2841).

Male C57BL/6N mice were purchased from Charles River Germany GmbH (Sulzfeld, Germany). The 5‐week‐old mice were acclimatized for 1 week at the local animal housing facility (Zentrales Tierlabor, Hannover Medical School). The mice were kept individually with retreats such as small houses in the cages in a temperature‐controlled environment (21 ±2°C) and had ad libitum access to food and drinking water. From the age of 6 weeks, they were then either fed a control diet (CD; 11 kcal% fat; S3542‐E040, ssniff Spezialdiäten, Soest, Germany) or a high‐fat diet (HFD; 60 kcal% fat; S3542‐E044, ssniff) for 30 weeks.

After 27 weeks, some mice of both diet groups were exposed to normobaric hypoxia (CD‐Hyp, HFD‐Hyp) in a hypoxia chamber (BioSpherix, Ltd., Parish, NY, USA) at 13% O_2_. The body weight and food consumption were recorded regularly. The different feeding and oxygen conditions resulted in the following groups: CD (*n* = 7), CD‐Hyp (*n* = 8), HFD (*n* = 8), HFD‐Hyp (*n* = 10). The animal cohort used in this study was also used for previous analyses (Mühlfeld et al., 2024; Pankoke et al., 2023).

### 2.2 Heart preparation and sampling

After 30 weeks, the mice were placed under deep anesthesia by intraperitoneal application of ketamine (100 mg/kg body weight; cp pharma, Burgdorf, Germany) and xylazine (5 mg/kg body weight; Bayer, Leverkusen, Germany). The thoracic cage was opened, and the heart was isolated. The heart was immersion‐fixed with 4% paraformaldehyde in phosphate buffer and kept in the fixative solution for at least 24 h. The RV was separated and weighed. Volumes of the heart and the isolated RV was determined by dividing the corresponding weight by the density of myocardial tissue (1.06 g/cm^3^; Mendez & Keys, 1961). Samples for light and transmission electron microscopic analysis were obtained by systematic uniform random sampling (SURS) of the RV (Schipke et al., 2017).

### 2.3 Processing of samples

Samples for light microscopy were embedded in paraffin according to a standard protocol. Briefly, paraffin embedding was carried out using the Citadel 1000 tissue processor (Shandon). After pre‐incubation in 70% isopropanol (2‐propanol, J.T. Baker, no. 8119) on the previous day, a standardised 24‐h programme was used. This comprised stepwise treatments: 3 h in 70% isopropanol, twice 2 h in 90% isopropanol, three times 2 h in 100% isopropanol, three times 2 h in methyl benzoate (Merck, no. 1.0659.2500), 1 h in Rotisol (Roth, no. 7917.1) and finally 6 h in Paraplast (Paraplast X‐TRA, Roth, no. X882.1). The samples were then moulded into blocks in paraffin. Paraffin sections of 4 μm thickness were generated for analysis.

For electron microscopy, samples were post‐fixed in 1.5% glutaraldehyde and 1.5% paraformaldehyde in 0.15 M HEPES buffer at a pH value of 7.35 for at least 24 h, washed, and incubated in 1% osmium tetroxide in 0.1 M sodium cacodylate buffer in the dark for 2 h. After washing, samples were incubated overnight in uranyl acetate at 4°C for block contrasting. After further washing and dehydration in an ascending acetone series, the tissue was incubated in an epoxy resin/acetone mixture for 1–3 h. The samples were then rotated overnight in 100% epoxy resin. The finished samples were transferred to flat‐bed moulds, aligned and hardened for 24 h at 40°C. From three randomly chosen blocks per animal, 60 nm ultra‐thin sections were cut and drawn onto copper grids coated with Formvar. The sections were contrasted with uranyl acetate and lead citrate.

### 2.4 Immunohistochemistry

For immunohistochemical analysis, sections were stained with the neuronal marker protein gene product (PGP) 9.5. This well‐established neuronal marker is particularly suitable for visualising nerve fibres in the cardiovascular system because of its high sensitivity for fine perivascular nerve fibres and its ability to accurately depict nerve structures (Gulbenkian et al., 1987).

Sections were deparafinized by xylol and a descending alcohol series. Heat‐induced epitope unmasking was achieved by microwaving the myocardial sections in Dako Retrieval buffer at pH 9. A peroxidase block (Santa Cruz Biotechnology, CAS 7722‐84‐1) was then performed to deactivate the endogenous peroxidase and prevent a subsequent reaction with DAB (3,3′‐diaminobenzidine) (Vector laboratories, SK‐4100). Avidin/biotin blocking was performed to reduce non‐specific binding and improve the specific detection of target molecules (Avidin/Biotin blocking kit, Vector laboratorys, VEC‐SP‐2001).

Incubation with the primary antibody (PGP 9.5 rabbit anti human protein gene product 9.5; polyclonal antibody IgG; BioTrend; no. BT78‐6305‐04; diluted to 1:500) was carried out overnight. After a short wash, the secondary antibody (rabbit IgG secondary antibody biotin conjugated preadsorbed, goat; polyclonal; Dianova; DNA‐SEC‐183348; diluted to 1:100) was applied to the sections for 1 h. The reaction was amplified using the ABC (avidin‐biotin complex) vector kit (Vector laboratoriys, VECTASTAIN Elite ABC‐HRP‐Kit, PK 6100). Finally, the section was stained with hemalum. After the staining process, the sections were dehydrated using an ascending alcohol series, covered with Roti‐Histokit 2 (Roth, T160.2) and sealed with a coverslip.

### 2.5 Stereology

Design‐based stereology was used for quantification as specified for the heart by Mühlfeld et al. (2010a). SURS was utilized whenever the amount of samples used for further analysis was reduced. The orientation of the samples was randomized for the light microscopic samples by the isector (Nyengaard & Gundersen, 1992) and for the electron microscopic samples by randomly dropping the samples in the embedding medium (Stringer et al., 1982). In line with previous studies, we relied on haphazard orientation during embedding (Schipke et al., 2014). The investigators (L‐C. M.; C.M.) were blinded from the beginning to the end of the stereological analysis to the group identity of the samples. The sections were analysed using a DM 6000B light microscope (Leica, Wetzlar, Germany) equipped with a DP72 digital camera (Olympus, Hamburg, Germany) and the NewCast programme (Visiopharm Integrator System (VIS) Version 3.6.5.0, Hørsholm, Denmark). For analysis, fields of view were obtained by SURS (Tschanz et al., 2014). Four unbiased counting frames were placed on the fields of view. Nerve fibres detected by PGP 9.5 staining were counted if they were fully or partly present in the counting frame area and did not touch the exclusion lines or their extensions. To assess the counting frame area used for counting, the upper right corner of each counting frame was counted if it hit heart tissue. In general, approximately 100–200 nerve fibre profiles were counted per animal.

The length density L_V_ of the nerve fibres in the RV can be estimated as follows using the general formula L_V_ = 2Q_A_. The number of counted nerve fibre profiles (Q(nf)) was divided by the total area of the counting frames (P(cf)) hitting the reference space, where a(p) is the counting frame area associated with one point, here 9084 μm^2^ (Mühlfeld et al., 2010b).

$$L_{v}\;\left(\mathrm{nf}/\mathrm{RV}\right)=\frac{\;2\times \sum Q\left(\mathrm{nf}\right)}{\left(\sum P\left(\mathrm{cf}\right)\times a\;\left(p\right)\right)}$$

To estimate the total length of the nerve fibres in the RV (L(nf, RV)), the length density of the nerve fibres (L_V_(nf/RV)) was multiplied by the volume of the RV (V(RV)). The resulting formula was therefore:

$$L\left(\mathrm{nf},\mathrm{RV}\right)=\frac{\;2\times \sum Q\left(\;\mathrm{nf}\right)}{\left(\sum P\left(\mathrm{CF}\right)\times a\left(p\right)\right)}\times V\left(\mathrm{RV}\right)$$

**FIGURE 1:**
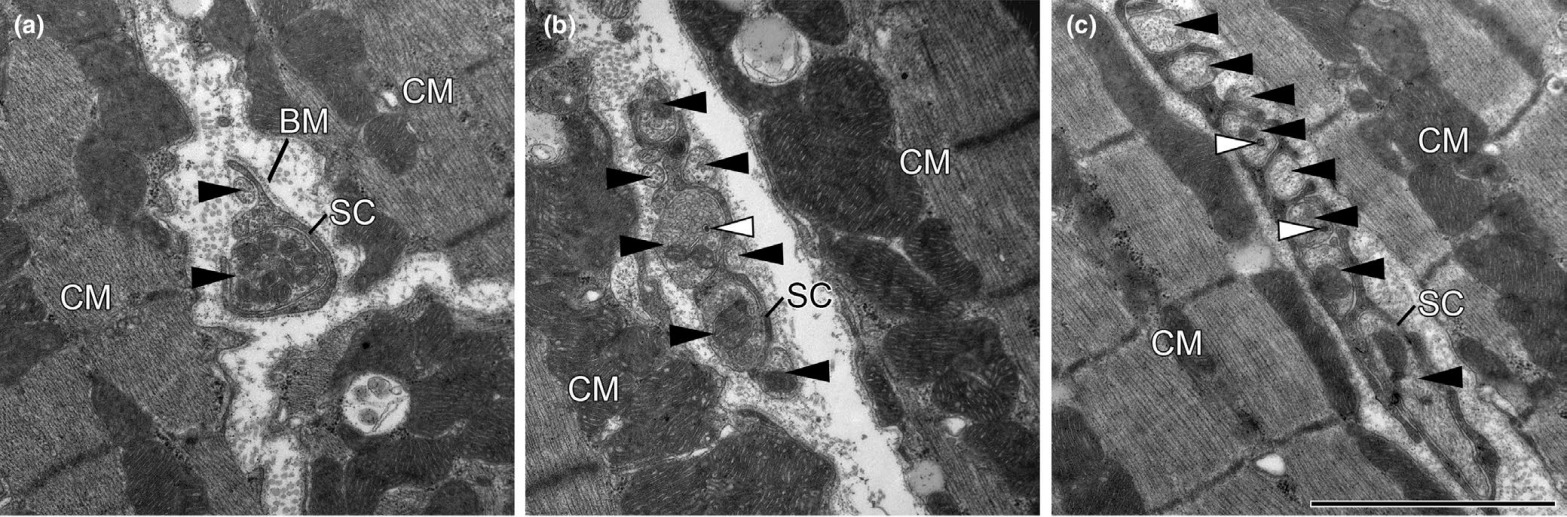
Ultrastructure of nerve fibres with different numbers of axons between cardiomyocytes. Electron micrographs were imaged at a primary magnification of 22,000×. Scale bar: 2 μm. black arrow heads, axon profiles; BM, basement membrane; CM, cardiomyocytes; SC, Schwann cell; white arrow heads, dense core vesicles.

**FIGURE 2:**
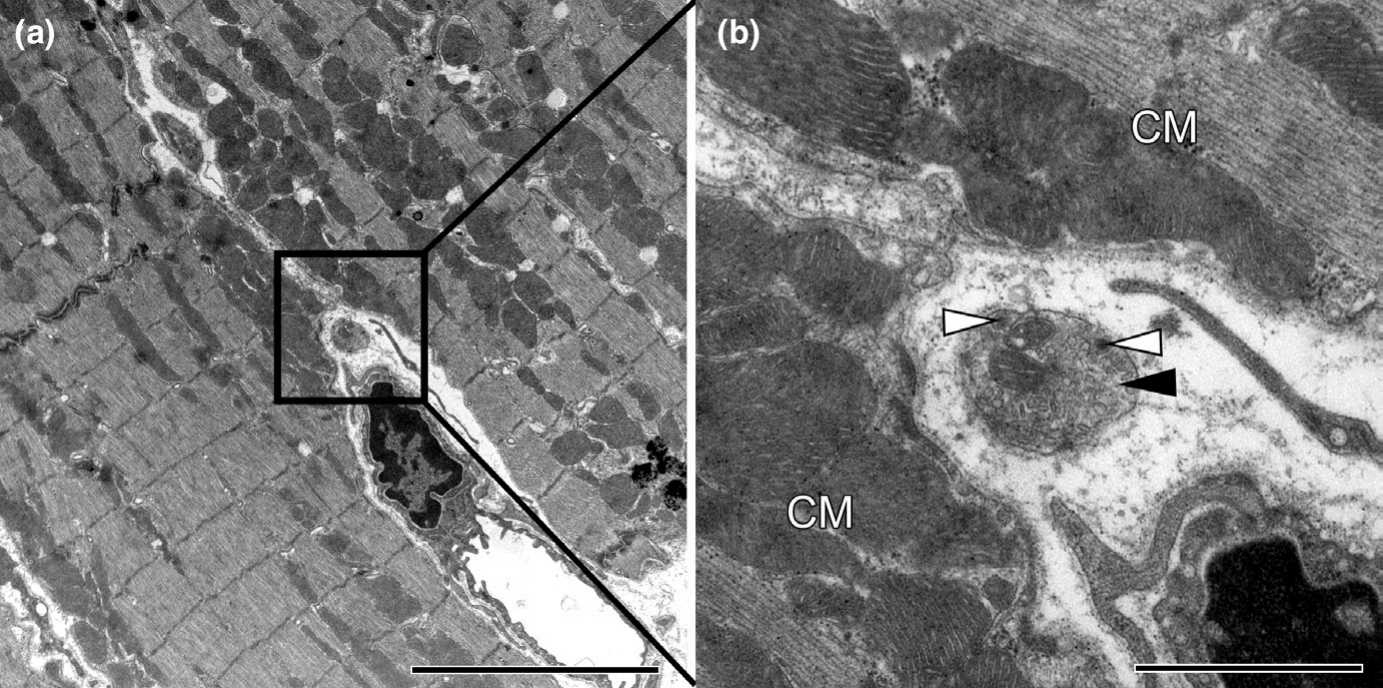
Ultrastructural overview of a small nerve fibre with a single axon between cardiomyocytes and a capillary. Electron micrographs were imaged at a primary magnification of 7100× (a) or 22,000× (b). Note that the axon is “naked”, that is, no Schwann cell process is visible. Scale bar in (a) 5 μm. Scale bar in (b) 1 μm. black arrow heads, axon profile; CM, cardiomyocytes; white arrow heads, dense core vesicles.

The transmission electron microscopic (TEM) analysis was carried out using a Morgagni 1 TEM (FEI, Eindhoven, The Netherlands) at a primary magnification of 22,000×. The specimens were subjected to SURS. Whenever a field of view contained a nerve fibre, images of the whole nerve fibre were taken. From these images, the number of axon profiles per nerve fibre profile were counted as well as the number of dense core vesicles. Representative TEM images showing the nerve fibres with varying numbers of axons between cardiomyocytes or between cardiomyocytes and capillaries are shown in Figures 1 and 2.

To determine the mean number of axon profiles per nerve fibre profile (Q_Q_(ax/nf)), the counted axon profiles (Q(ax)) were divided by the nerve fibre profiles (Q(nf)) according to the following formula:

$$Q_{Q}\left(\mathrm{ax}/\mathrm{nf}\right)=\frac{\sum Q\left(\mathrm{ax}\right)}{\sum Q\left(\mathrm{nf}\right)}$$

The total length of the axons (L(ax, RV)) was calculated as follows:

$$L\left(\mathrm{ax},\mathrm{RV}\right)=Q_{Q}\left(\mathrm{ax}/\mathrm{nf}\right)\times L\left(\mathrm{nf},\mathrm{RV}\right)$$

In the original study describing the stereological method of axon length estimation (Mühlfeld et al., 2010a) it was shown that the estimates from the paraffin sections are prone to tissue deformation. Correction for tissue shrinkage led to slightly lower values for the length of nerve fibres compared to calculations without this correction. Gruber et al. (2012a) showed that tissue shrinkage is equal in hearts of control or high‐fat diet fed mice. Therefore, in the present study we relied on equal shrinkage in the different experimental groups, thus accepting a slight overestimation of total nerve fibre and axon length in all groups.

The ratio of dense core vesicles per axon profile was calculated by dividing the number of dense core vesicles (Q(DCV)) by the number of axon profiles (Q(ax)).

To determine the surface density of the cardiomyocytes of the RV, electron microscopic images at a primary magnification of 1800× were analysed (Mühlfeld et al., 2024). The analysis was performed using the STEPanizer programme (version 2.28) (Tschanz et al., 2011). Twenty images of three blocks per animal were analysed. A line grid with eight test lines and a subsampling of 1:4 was used for quantification. The end points of the lines (P(myo)) were counted if they hit myocardium. Intersections (I(cm)) of the line segments with the cardiomyocyte membrane were also counted. The length of the test lines associated with one point (l(p)was 6.71 μm. The following formula was used to calculate the surface density (S_v_(mem/RV)):

$$S_{v}\left(\mathrm{mem}/\mathrm{RV}\right)=\frac{2\times \sum I\left(\mathrm{cm}\right)}{4\times l(p)\times \sum P\left(\mathrm{myo}\right)}$$

The absolute surface area was calculated from this according to:

$$S\left(\mathrm{mem},\mathrm{RV}\right)=S_{v}\left(\mathrm{mem}/\mathrm{RV}\right)\times V\left(\mathrm{RV}\right)$$

To determine the relationship between the nerve fibre length and the cadiomyocyte surface area, the ratio of the total length of the nerve fibres and the absolute surface area of the cardiomyocytes was calculated.

Similarly, the relationship between the axon length and the surface area of the cardiomyocytes was calculated by dividing the total length of the axons by the total surface area of the cardiomyocytes.

### 2.6 Statistics

Statistical analysis was performed using SigmaPlot® software version 13.0 (SYSTAT®Software Inc.). The data were tested for normality using the Kolmogorov–Smirnov test and for equal variance using the Brown‐Forsythe test. In cases where the normality test failed, the data were ln transformed (here: length density L_V_; axon profiles per nerve fibre profile Q_Q_(ax/nf)). Subsequently, a two‐way ANOVA followed by a post‐hoc Tukey test was performed. In line with the Guidelines of the American Physiological Society for reporting statistics (Curran‐Everett & Benos, 2004), data were regarded as significantly different if *p* < 0.05 and suggestive of a difference, that is, a tendency if 0.05 < *p* < 0.1.

Means and standard deviations were calculated. The data are presented as individual values of each animal in the form of symbols (CD○; CD‐Hyp●;HFD□;HFD‐Hyp■) with means indicated by horizontal bars and standard deviation in green or as numerals in the tables. GraphPad Prism (version 7) was used for data visualization.

## 3 RESULTS

### 3.1 Animal model

As reported previously (Mühlfeld et al., 2024; Pankoke et al., 2023), body weight was similar between the normoxic and hypoxic animals fed CD, but HFD caused higher body weights in both experimental groups compared to their respective control groups. However, hypoxia within the HFD intervention led to weight loss, possibly due to 20% decreased calorie consumption. Both hypoxia and HFD caused RV hypertrophy with an additive effect of both interventions together. The RV‐to‐body weight ratio shows that RV hypertrophy was proportional to body weight under HFD but significantly increased under hypoxia (Table 1).

**TABLE 1:** Summary of data.

Diet	Control diet (lean)		High‐fat‐diet (obese)	
Exposure	Normoxia	Hypoxia	Normoxia	Hypoxia
*n*	7	8	8	10
Body weight [g]	37.23 ± 2.99	34.47 ± 2.86	49.70 ± 3.63	42.22 ± 4.10
RV Weight [g]	0.017 ± 0.003	0.023 ± 0.004	0.023 ± 0.005	0.028 ± 0.004
RV Volume [mm^3^]	16.44 ± 2.43	21.70 ± 3.49	21.46 ± 4.70	25.94 ± 4.01
L_V_(nf/RV) [x 10^‐3^ μm^−2^]	0.158 ± 0.04	0.176 ± 0.06	0.206 ± 0.054	0.147 ± 0.05
L(nf, RV) [m]	2.61 ± 0.77	3.80 ± 1.26	4.37 ± 1.51	3.74 ± 0.96
L(ax, RV) [m]	8.87 ± 2.75	11.54 ± 3.16	15.95 ± 4.62	10.90 ± 2.45
Q_Q_(ax/nf)	3.44 ± 0.68	3.12 ± 0.44	3.73 ± 0.54	2.95 ± 0.43
DCV/Q(ax)	0.38 ± 0.111	0.38 ± 0.091	0.34 ± 0.077	0.31 ± 0.059
RV‐to‐Body weight ratio [x10^6^]	468 ± 59	666 ± 85	461 ± 113	654 ± 98
S_V_(mem/RV) [μm^−1^]	0.178 ± 0.037	0.158 ± 0.049	0.177 ± 0.030	0.175 ± 0.020
S(mem, RV) [cm^2^]	29.71 ± 9.03	34.33 ± 13.30	37.28 ± 7.29	45.51 ± 8.94
L(ax, RV) /S(mem, RV) [m/cm^2^]	0.319 ± 0.122	0.391 ± 0.210	0.448 ± 0.178	0.243 ± 0.056

*Note*: Values are given as mean values ± SD. Statistics: two‐way ANOVA (factors diet and hypoxia), followed by post hoc Tukey test; significant differences are displayed in Figure 4. Body weight, weight of RV, volume of RV, RV‐to‐Body weight ratio have already been published in previous studies (Mühlfeld et al., 2024; Pankoke et al., 2023).

Abbreviations: DCV/Q(ax), mean number of dense core vesicles per axon profile; L(ax, RV), total length of axons in RV; L(nf, RV), total length of nerve fibres in RV; L_V_(nf/RV), length density of nerve fibres per unit volume of RV; Q_Q_(ax/nf), mean number of axon profiles per nerve fibre profile; RV, right ventricle; S(mem, RV), total surface area of sarcolemma in RV; S_V_(mem/RV), surface density of sarcolemma per unit volume of RV.

### 3.2 Innervation

**FIGURE 3:**
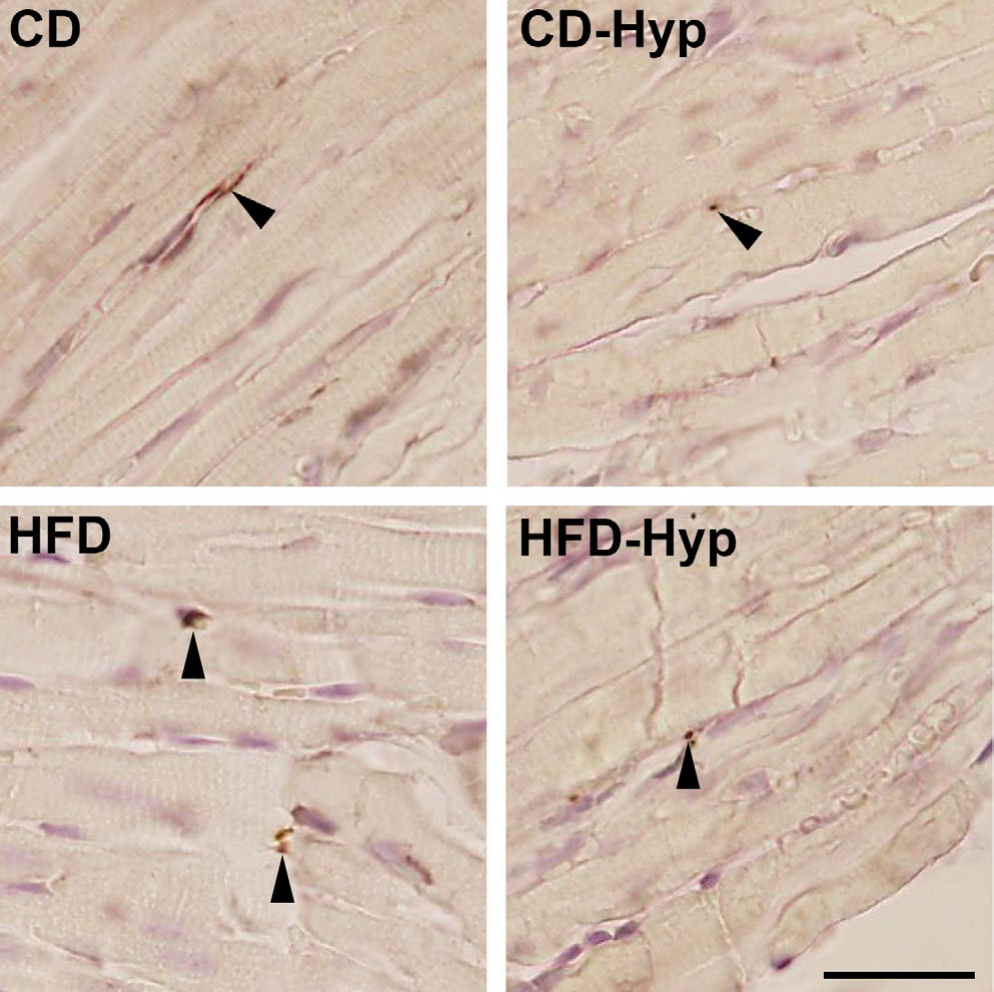
PGP 9.5 labelling of nerve fibres. Light micrographs were imaged at an objective lens magnification of 40×. Scale bar 25 μm. Arrows = PGP 9.5 labeled nerve fibres.

Nerve fibres were frequently detected in PGP 9.5 stained paraffin (Figure 3) and ultrathin epoxy resin (Figures 1 and 2) sections in all groups. Larger nerve fibres were mainly present near blood vessels whereas nerve fibres between cardiomyocytes often were smaller and contained a fewer number of axons. As presented in Figure 2, nerve fibres consisting of single axons were sometimes devoid of Schwann cell processes. Thus, using the stereological approach of length estimation, the total length of nerve fibres, the mean axon profiles per nerve fibre as well as the total length of axons innervating the RV could be compared.

**FIGURE 4:**
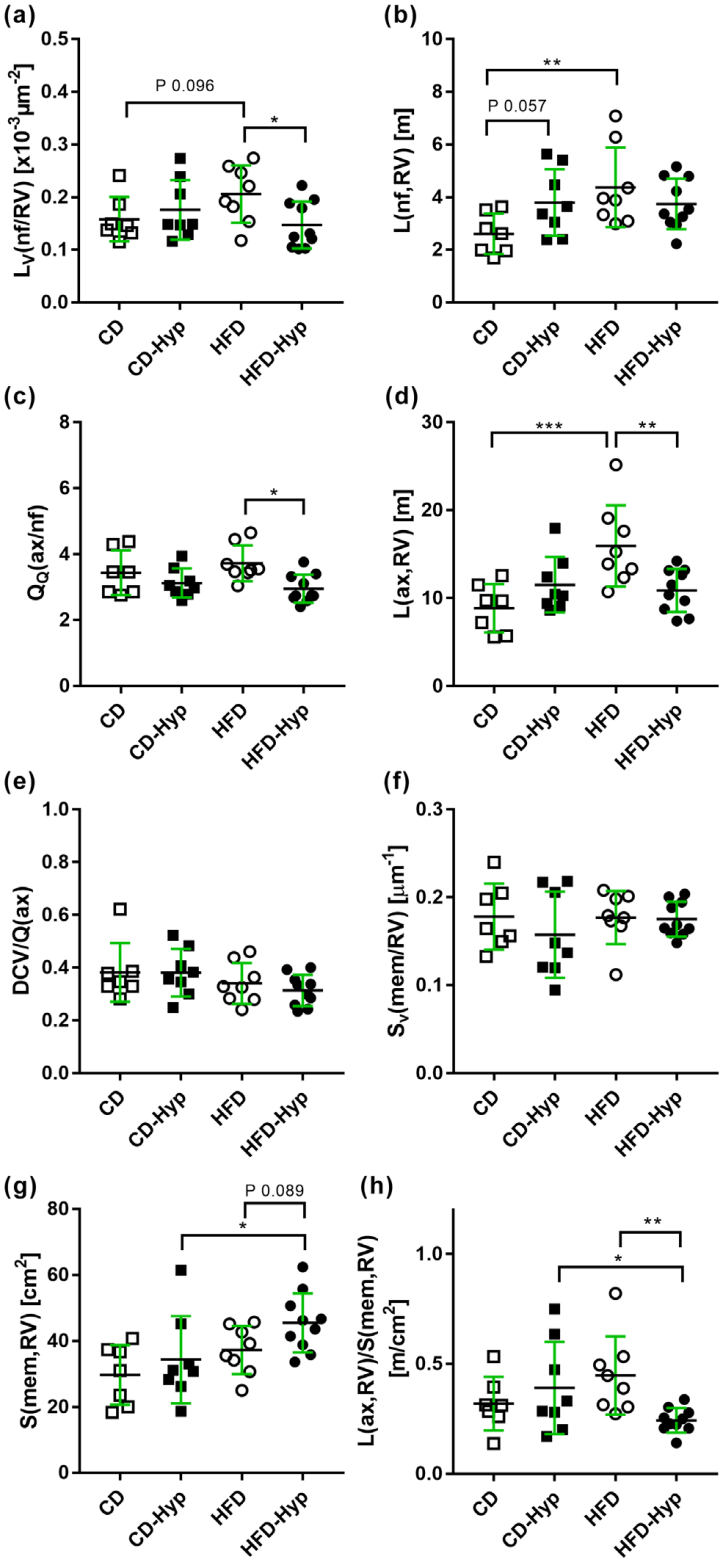
Stereological results. Each data point represents one animal. Length density of nerve fibres (a); total length of nerve fibres (b); axon profiles per nerve fibre profile (c); total length of axons (d); dense core vesicles per axon profile (e); surface area of cardiomyocytes (f); total surface of cardiomyocytes (g); total length of axons per cardiomyocyte membrane (h). Data are presented either as individual values of each animal in the form of dots/boxes or as mean value of the group represented by horizontal bars. Standard deviation is displayed in green. CD, control diet under normoxia; CD‐Hyp, control diet under hypoxia; HFD, high‐fat diet under normoxia; HFD‐Hyp, high‐fat diet under hypoxia. Two‐way ANOVA followed by post hoc Tukey test. Significant differences indicated *p < 0.05; **p < 0.01;***p < 0.001 or in the case of a tendency as precise *p* value.

The relative nerve fibre length density was similar in CD and CD‐Hyp. A trend towards a higher length density was observed in the HFD group compared to the control group. In HFD‐Hyp the length density was significantly smaller than in HFD (Figure 4a). The total length of nerve fibres was significantly higher in mice fed a HFDcompared to the control group, showing an increase of 67.4%. In contrast, the CD‐Hyp group exhibited only a trend towards higher nerve fibre length in comparison with CD (*p* = 0.057). No significant differences were observed between the other groups (Figure 4b). Interestingly, the mean number of axon profiles per nerve fibre profile was significantly smaller in HFD‐Hyp than in HFD while no changes were observed in the other groups (Figure 4c).

The total length of axons in the RV did not show significant differences between CD and CD‐Hyp. In contrast, HFD resulted in an 80% increase in total axon length compared to the control group. Contrary to the hypothesis, the total length of axons in HFD plus hypoxia was significantly lower than the HFD group alone (Figure 4d). Thus, no synergistic or additive effect of HFD and hypoxia was observed. No significant differences were observed in the number of dense core vesicles per axon profile among the experimental groups (Figure 4e).

### 3.3 Cardiomyocytes

The surface area density of the cardiomyocyte plasma membrane was similar in all groups (Figure 4f). The total surface area of the cardiomyocytes was similar in CD, CD‐Hyp and HFD. In HFD‐Hyp, this parameter was significantly elevated by 32.6% compared with CD‐Hyp and tended to be higher than in HFD (Figure 4g). Thus, the relationship between total axon length and cardiomyocyte surface area was similar among CD, CD‐Hyp and HFD but smaller in HFD‐Hyp by 23.8%, 38% or 45.8% compared to CD, CD‐Hyp and HFD, respectively (Figure 4h).

## 4 DISCUSSION

This study presents the first quantitative stereological analysis of nerve fibres in the RV using a clinically relevant model combining obesity induced by a HFD with chronic hypoxia.

In line with the hypothesis, the length density of nerve fibres, the total length of nerve fibres as well as the total length of axons ramifying between cardiomyocytes was significantly enhanced in mice fed HFD under normoxia. In contrast to our initial hypothesis, however, the combination of hypoxia with HFD resulted in a lower length density of nerve fibres, a lower number of axon profiles per nerve fibre profile and a lower total length of axons than in HFD under normoxic conditions.

For interpretation of the stereological data, it is important to distinguish between relative and total values and for a full picture it is useful to look at both of them. Relative values, such as length density of nerve fibres, alone are difficult to interpret as they can be influenced by changes in the numerator and the denominator. However, an increase in length density also proves that a single nerve fibre supplies a smaller area of myocardium which may have functional consequences. In contrast, it is important to look at the total length to see whether there has been a true increase in nerve fibre length, that is, that new biological structures have been built. Both relative and total values, however, may either be direct effects of the experimental condition, a secondary effect due to the myocardial changes caused by the experimental condition or a secondary effect due to some other effect caused by the experimental condition.

In the animal model used in the present study, the weight of the RV was enhanced proportional to the body weight increase induced by HFD. Body and heart weight are closely correlated with each other, and physiological hypertrophy of the heart occurs as an adaptation to increased metabolic demands (Eslami et al., 2017; Lindstedt & Calder, 1981; Ortega et al., 2016; Schipke et al., 2014). Chronic hypoxia is a hypertrophic stimulus that surpasses the HFD effect as shown by the ratio of RV weight to body weight. The hypoxic exposure had a similar effect on the increase in RV weight and volume independent of the body weight. In a previous study, chronic hypoxia caused remodelling of pulmonary vessels in the current animal model (Pankoke et al., 2023), probably due to vasoconstriction of the pulmonary vessels which in turn induces increased pressure on the RV and PH. Therefore, the current animal model combines features of HFD‐induced and chronic hypoxia‐induced hypertrophic response of the RV.

The present study was conducted exclusively on male mice, thus limiting the transferability and generalizability of the results to both sexes. According to a study by Neupane et al. (2020), in lean and obese rats of both sexes with Monocrotaline‐induced PH, sex differences were observed in the severity of the disease, with overweight female rats experiencing more severe symptoms than their lean controls. In contrast, no significant sex differences in disease severity were found in lean and obese mice with hypoxia‐induced PH.

In addition to heart growth, several experimental studies have shown that obesity is associated with changes in the ANS (Aubin et al., 2010; Grassi et al., 2015; Silvani et al., 2010). Evidence shows a clear link between elevated blood pressure and sympathetic activation (Grassi, 2009; Grassi et al., 2023; Kondo et al., 1996). In addition, hyperinsulinemia, which can occur in obesity, also leads to increased sympathetic activity (Anderson et al., 1992). Gruber et al. (2012a) used a similar animal model of diet‐induced obesity and the same stereological approach as in the present study to investigate the innervation of the LV. Notably, the nerve fibre length density of the control group closely matches the values observed in this study, indicating a similar amount of nerve fibres per unit volume of the RV. However, the number of axons per nerve fibre profile was higher in the present study than in the LV described by Gruber et al. (2012a). As a consequence, the total length of the axons in the RV was nearly half as high as in the LV indicating a denser innervation of the RV myocardium. In obese mice, the present study also detected a slightly higher nerve fibre length density compared to Gruber et al. (2012a), along with a greater number of axons per profile, thus leading to the diet‐induced significant increase in total axon length in HFD compared to CD. Gruber et al. (2012a) did not detect a diet‐induced increase in total axon length in the LV. This difference may reflect regional variations in left and right ventricular adaptation, however, Gruber et al. only used *n* = 5 animals per group which may have hindered the data from showing a statistically significant difference.

In a cancer cachexia mouse model with cardiac atrophy, the stereologically estimated total axon length was reduced in the atrophic left ventricles of tumor‐bearing mice compared with the control animals (Mühlfeld et al., 2011). In that study, it was hypothesised that the reduced nerve growth factor (NGF) levels associated with cachexia were responsible for the hypoinnervation. NGF expression has a strong influence on the regulation of the sympathetic innervation of the left ventricle (Cao et al., 2000; Kaye et al., 2000). Longer and denser nerve fibres were observed in experimental models with increased NGF concentration (Hassankhani et al., 1995). Kiriazis et al. (2005) demonstrated that mice with cardiac overexpression of NGF developed marked sympathetic hyperinnervation, accompanied by hypertrophy and fibrosis in the RV. It would be tempting to speculate that in obesity NGF overexpression causes hyperinnervation, however, evidence suggests that in obesity‐related metabolic disorders NGF and brain derived neurotrophic factor signaling is reduced (Frohlich et al., 2021).

Chronic hypoxia can lead to PH (Hautbergue et al., 2021; Semenza, 2012) as demonstrated by several clinical and experimental studies, which also suggests that this condition results in increased sympathetic activity (Velez‐Roa et al., 2004). Surprisingly, the results in obese mice under hypoxic conditions showed reverse effects compared to normoxic obese mice. While hypoxia did not influence the measured parameters in lean mice, the combination of hypoxia and obesity resulted in a reduction in both the length density of nerve fibres and the total length of axons. This contrasts with previous studies that showed increased sympathetic activation under chronic hypoxia (Calbet, 2003; Swenson, 2020). Velez‐Roa et al. (2004) reported that this increased sympathetic activity is partly mediated by the chemoreflex and can be reduced by the administration of 100% oxygen, indicating chronic hypoxia as the driving factor for sympathetic hyperexcitation. Ruijtenbeek et al. (2000) demonstrated in a chicken embryo study that chronic hypoxia contributes to sympathetic hyperinnervation, resulting in increased density of periarterial nerve fibres and elevated noradrenaline content in the heart. These findings suggest that hypoxia can induce sympathetic activation, which over time may contribute to endothelial dysfunction, inflammation and oxidative stress, ultimately increasing the risk of cardiovascular disease (Da Dalt et al., 2023; Swenson, 2020). As in the present animal model hypoxia did not cause RV hyperinnervation in lean animals and even reversed HFD‐induced hyperinnervation, it has to be taken into account that the functional properties of the ANS not only depend on the structural properties but also on number of varicosities and transmitter expression. As shown here, at least for the mean number of dense core vesicles per axon profile no difference was observed among the groups in this study.

One potential explanation for the decreased axon length in HFD‐Hyp compared with HFD could be related to the hypoxia‐induced weight reduction. Previous studies have shown that the sympathetic overexcitation caused by obesity can be reduced by a hypocaloric diet (Grassi et al., 1998; Straznicky et al., 2005, 2010). After a 12‐week lifestyle intervention, significant weight loss in subjects with metabolic syndrome led to a reduction in resting sympathetic activity, as evidenced by decreased norepinephrine spillover rates. Additionally, an increased sympathetic response to glucose was observed, suggesting improved central insulin sensitivity and enhanced sympathetic reactivity (Straznicky et al., 2009).

Caloric restriction did not result in differences of total axon length in the left ventricle of the mouse heart, but caloric restriction was only performed in lean control, not in obese mice (Gruber et al., 2012b). In this study, the HFD‐fed mice under chronic hypoxia consumed 20% less calories than the HFD‐fed mice under normoxia and exhibited a significant weight loss. Thus, this weight loss may potentially explain the reduction of nerve fibre length density and total axon length in the HFD‐Hyp group.

From a functional perspective, the observed morphological changes may affect the cardiac capacity to modulate its function according to mechanical or metabolic needs. Increased innervation as displayed by higher nerve density may therefore cause a sympathetic hyperactivity. Thus, the effect of therapeutics influencing the sympathetic signalling, such as beta blockers, may be influenced by the morphological characteristics of the myocardial innervation.

In summary, diet‐induced obesity led to hyperinnervation of the RV in the male mouse, but these effects were partially reversed under hypoxia, indicating a complex interaction between hypoxia, obesity and the sympathetic innervation. The mechanisms behind these alterations and the functional impact of the structural changes require further analysis.

## AUTHOR CONTRIBUTIONS

Louisa‐Chiara Mierswa contributed to acquisition of data, data analysis/interpretation, drafting of the manuscript. Julia Schipke contributed to concept/design and data analysis/interpretation. Christian Mühlfeld contributed to concept/design, data analysis/interpretation and drafting of the manuscript. All authors critically revised the manuscript and approved the final version of the article.

## CONFLICT OF INTEREST STATEMENT

The authors declare no conflicts of interest.

## Data Availability

The data that support the findings of this study are available from the corresponding author upon reasonable request.
